# An investigation of the factors influencing discharge readiness in colorectal cancer stoma patients

**DOI:** 10.1097/MD.0000000000047220

**Published:** 2026-01-30

**Authors:** ShuHan Li, KaiXin Liu, WenNv Hao

**Affiliations:** a Orthopedics B Area, Inner Mongolia Medical University Affiliated Hospital, Hohhot, Inner Mongolia, China; b Shanxi Provincial People’s Hospital, Taiyuan, Shanxi, China; c Department of Emergency, Affiliated Hospital of Inner Mongolia Medical University, Hohhot, Inner Mongolia, China.

**Keywords:** colorectal cancer, factor analysis, patient discharge, stoma

## Abstract

This study aims to understand the discharge readiness status of colorectal cancer stoma patients and analyze their influencing factors to provide a basis for healthcare professionals to develop discharge guidance intervention strategies. This study adopted a cross-sectional study design. A convenience sampling method was used to select patients with colorectal cancer stoma in 2 tertiary-level hospitals in Hohhot, Inner Mongolia Autonomous Region, from August 2023 to January 2025. A general information questionnaire, Discharge Guidance Quality Scale, and discharge readiness scale for patients with colorectal stoma were used to conduct the survey. One-way analysis and multivariate stepwise linear regression were applied to analyze the influencing factors of the patients’ discharge readiness, with the level of significance set at *P* < .05 in the statistical analysis. The discharge readiness score of colorectal cancer stoma patients was (119.53 ± 29.14), which was at a moderate level, and the scores of each dimension were in descending order of expected support, disease knowledge, personal status, and coping ability. The results of multivariate stepwise linear regression analysis showed that the quality of discharge instructions, postoperative complications, and place of residence were factors influencing the discharge readiness of colorectal cancer stoma patients (*P* < .05). The discharge readiness of colorectal cancer stoma patients is moderate and requires further improvement. Among them, the quality of discharge instructions, postoperative complications, and place of residence are all key factors influencing the discharge readiness of colorectal cancer stoma patients.

## 1. Introduction

Colorectal cancer (CRC) is a malignant lesion originating from the mucosal epithelium of the colon or rectum, and is an important component of gastrointestinal tumors. Currently, the main treatment for CRC is combination therapy with surgery as the core treatment, supplemented by various therapies such as radiotherapy, chemotherapy, and molecular targeted therapy.^[1]^

With the rapid development of medical technology, although anus-preserving surgery can meet the therapeutic needs of most CRC patients, many CRC patients inevitably need to have a temporary or permanent enterostomy to solve the problem of defecation, reduce the possibility of cancer, or prevent multiple postoperative complications.^[2]^

Studies have shown that the number of patients undergoing enterostomy surgery in China is as high as 100,000 annually. According to statistics, the total number of patients with intestinal stoma in China exceeds 1 million.^[3]^

According to the Action Plan for Comprehensively Improving the Quality of Medical Care (2023–2025) issued by the National Health Commission, healthcare organizations should comprehensively strengthen the assessment of patients at key points in time, such as perioperative and predischarge, to ensure that the assessment is standardized and systematic, and to expand the coverage of quality care services.

Discharge readiness was proposed by Prof Fenwick in the 1970s and has become increasingly important as an indicator for assessing whether patients can leave the hospital safely and smoothly, reintegrate into society, and complete subsequent rehabilitation. Good readiness for discharge not only helps to reduce the rate of unplanned readmission and improve the quality of life of patients but also promotes the rational use of healthcare resources and reduces overall healthcare costs.^[4]^

Especially in the context of the current healthcare environment, hospitals are actively taking measures to effectively reduce the average length of stay,^[5]^ and the field of rectal surgery is no exception, with the combination of multidisciplinary team, perioperative medicine, and enhanced recovery after surgery. Surgery has been effective in shortening the average length of stay,^[6]^ as well as shortening the time patients spend in the hospital receiving treatment and care, and increasing the difficulty of transitioning from hospital care to home care at discharge.

Discharge readiness, as an assessment of whether patients have the ability to independently leave the hospital and effectively manage their own health conditions, is particularly important for the recovery process of patients with stoma.

This study aimed to understand the discharge readiness status of CRC stoma patients and analyze their influencing factors to provide a basis for healthcare professionals to develop discharge guidance intervention strategies.

## 2. Methods

### 2.1. Type of study

This study adopts a cross-sectional descriptive design. It aims to explore the current status of discharge guidance quality and discharge readiness among inpatients with CRC stoma, and analyze the influencing factors of discharge readiness. The study design and reporting comply with the guidelines of the Strengthening the Reporting of Observational Studies in Epidemiology checklist, ensuring consistency between the study type and subsequent statistical analyses.

### 2.2. Study subjects

#### 2.2.1. Source of subjects

This study was conducted in the inpatient departments of 2 tertiary-level general hospitals in Hohhot City, Inner Mongolia Autonomous Region, China. Tertiary-level hospitals were selected as the research sites because they are regional medical centers with comprehensive diagnostic and treatment capabilities for CRC, admitting a large volume of patients who undergo colorectal stoma surgery – ensuring access to a representative sample of the target population (CRC patients). Within these hospitals, the study specifically focused on 3 clinical departments directly involved in the diagnosis, treatment, and postsurgical care of CRC stoma patients: the Emergency Surgery Department, Gastrointestinal Surgery Department, and Abdominal Surgery Department. These departments were chosen due to their primary responsibility for managing patients who require colorectal stoma placement and their role in delivering perioperative and discharge-related care, which aligns with the study’s focus on discharge readiness. Detailed hospital names are not disclosed to protect institutional privacy, but both facilities meet national standards for tertiary hospitals in China, with:

1. dedicated gastrointestinal oncology units and stoma care clinics;
2. multidisciplinary teams (surgeons, nurses, stoma therapists, and nutritionists) experienced in managing CRC stoma patients;
3. standardized perioperative care protocols and discharge guidance procedures, which ensured consistency in the care context for enrolled patients.

#### 2.2.2. Inclusion and exclusion criteria

Inclusion criteria: patients with pathologically confirmed CRC who underwent colostomy; aged ≥ 18 years; and with clear consciousness, able to independently complete or assist in completing questionnaires and scales (e.g., no severe visual or hearing impairment that affects information acquisition).

Exclusion criteria: complicated with damage to important organs (heart, liver, kidney, etc) or other severe chronic diseases; with confirmed tumor metastasis (e.g., liver metastasis and lung metastasis) via imaging or pathological examination; and accompanied by mental disorders or cognitive dysfunction.

#### 2.2.3. Sample size calculation and recruitment outcome

Sample size estimation: according to Kendall M’s sample estimation method,^[7]^ the sample size should be 10 to 20 times the number of variables. This study included 17 variables in total: 14 variables from the general information questionnaire and 3 variables from the Discharge Guidance Quality Scale.

Adjustment for failure rate: considering a 10% potential data failure rate and the stability of the statistical model, the initial sample size was set to 188 to 378 cases.

Recruitment and screening results: during the study period, a total of 243 patients were screened. Among them, 12 patients were excluded due to unqualified data or noncooperation: specifically, 3 cases of questionnaire missing answers (key items such as tumor location or medical payment method were unfilled, and supplementary verification could not be completed), 7 cases of noncooperation in telephone follow-up surveys (refused to complete the remaining scale items through telephone after preliminary on-site communication), and 2 cases of identical answers for all options (suspected of perfunctory filling, with no logical differences between scale items, thus judged as invalid); no patients refused to participate in the study at the initial consent stage.

Valid sample confirmation: finally, 231 valid samples were included, which met the estimated sample size requirement.

#### 2.2.4. Ethical approval

This study was reviewed and approved by the Ethics Committee of the Affiliated Hospital of Inner Mongolia Medical University (approval number: YJS2025014). All study procedures were conducted in accordance with the Declaration of Helsinki, and the right to know and privacy of patients were fully protected.

### 2.3. Research tools

#### 2.3.1. General information questionnaire

A general information questionnaire was designed on our own through a literature review, which included gender, age, ethnicity, marital status, work status, place of residence, mode of residence, education level, per capita monthly household income, mode of medical payment, number of days in hospital, tumor site, nature of the stoma, and postoperative complications, for a total of 14 entries.

#### 2.3.2. Quality of Discharge Teaching Scale

The instrument used in this study to measure quality of discharge teaching in CRC stoma patients was the Chinese version of the Quality of Discharge Teaching Scale by Wang et al.^[8]^ Chinese version of the Quality of Discharge Teaching Scale consists of 3 dimensions with a total of 24 entries: what patients self-perceived they needed before discharge (6 entries), what patients actually received before discharge (6 entries), and guidance skills and effects (12 entries). The Cronbach’s coefficient of the whole scale was 0.924, the content validity index (CVI) of each entry was 0.89 to 1.00, and the average CVI of all the entries was 0.98. The scale has good reliability and validity and is suitable for the evaluation and measurement of quality of discharge teaching in the Chinese cultural context. Scores of 0 to 10 were assigned from “not at all/not at all able” to “very much/always able,” with scores between 0 and 7 defined as insufficient, 7 and 8 as moderate, 8 and 9 as high, and 9 or more as very high quality of discharge teaching.^[9]^ The higher the score, the better the patient’s Quality of Discharge Teaching Scale score.

#### 2.3.3. Readiness for Hospital Discharge Scale for patients with enterostomies

Readiness for Hospital Discharge Scale developed by Yu^[10]^ was used in this study, which consists of 4 dimensions, with a total of 18 entries: personal status (3 entries), knowledge of the disease (8 entries), coping ability (3 entries), and anticipatory support (4 entries). Cronbach’s alpha coefficient of the scale was 0.923, and the Cronbach’s alpha coefficients of the 4 dimensions of personal status, knowledge of illness, coping ability, and anticipatory support were 0.806, 0.876, 0.844, and 0.947, respectively. The Item-level content validity index of the scale was 0.824 to 1 (>0.78) and that of the scale was 0.969. the scale has good The scale has good reliability and validity, and is suitable for the evaluation and measurement of discharge readiness in enterostomy patients in the Chinese cultural context. Each entry was assigned a score of 0 to 10, with a total score of 180. The scores were divided into 3 levels: 0 to 60 points, low level; 61 to 120 points, medium level; and 121 to 180 points, high level.

### 2.4. Data collection

#### 2.4.1. Preparation for data collection

Before data collection, the research team obtained written consent from the medical administration departments and nursing units of the 2 participating hospitals. All researchers received unified training to master the filling standards of the questionnaire, the explanation method of scale items, and the communication skills with special groups (such as elderly or low-literacy patients). For patients who could not complete the questionnaire on-site because of physical discomfort, a telephone follow-up survey plan was formulated in advance, specifying the follow-up time window (within 24 hours after the patient’s condition stabilized) and communication scripts to ensure the consistency of survey processes.

#### 2.4.2. Collection methods and tools

Questionnaire form: only paper questionnaires were used in this study. The questionnaires were uniformly printed on A4 paper, with clear fonts and reasonable spacing to reduce reading difficulty for elderly patients.

Survey process was as follows. On-site survey: for patients who could fill out independently, researchers explained the study purpose and requirements, then let patients complete the questionnaire on their own; for special groups, researchers read items aloud in a neutral tone, filled in answers according to patients’ true expressions, and avoided guiding language. Telephone follow-up: for patients who suspended on-site filling because of physical discomfort, researchers conducted follow-up within the scheduled time, verified the patient’s identity, 1st, then completed the remaining items, and repeated key answers to confirm accuracy.

Data verification: after the questionnaire was completed (either on-site or via telephone), researchers checked the paper questionnaire on the spot for 2 aspects: completeness and logical consistency. Questionnaires that met the standards were numbered sequentially, and stored in a locked file cabinet (with a dedicated person responsible for management) to ensure data security.

#### 2.4.3. Collection outcome

A total of 243 paper questionnaires were distributed, including 18 cases that required telephone follow-up to complete. After verification, 3 cases were excluded due to questionnaire missing answers (key items such as “tumor location” or “per capita monthly household income” were unfilled, and patients could not be reached for supplementation after 3 follow-up attempts); 7 cases were excluded due to noncooperation in telephone follow-up surveys (patients refused to continue answering after 1 to 2 attempts, or showed obvious impatience and provided answers that were inconsistent with clinical records); 2 cases were excluded due to identical answers for all options; and finally, 231 valid paper questionnaires were recovered, with an effective recovery rate of 95.06%.

### 2.5. Statistical methods

#### 2.5.1. Data management

Database establishment: 1st, an Excel 2021 raw data database was established, with each column representing a variable and each row representing a sample. The variable names were standardized (e.g., “gender_1 = male, gender_2 = female”) to avoid ambiguity.

Data entry and verification: for electronic questionnaires, data were automatically synchronized to the Excel database after the survey; for paper questionnaires, 2 researchers entered the data independently using the “double-entry method.” After entry, the 2 datasets were compared and verified. If there were differences, the original paper questionnaire was checked to ensure data accuracy. No data were altered during the entire process.

#### 2.5.2. Definition of variables

Outcome variable: the total score of the Readiness for Hospital Discharge Scale was used as the primary outcome variable, reflecting the level of discharge readiness of patients with CRC stoma.

Independent variables: Included general information variables (14 items; e.g., age, education level, and length of hospital stay) and the total score of the Quality of Discharge Teaching Scale (reflecting the quality of discharge guidance).

#### 2.5.3. Statistical analysis

Statistical analysis was performed using SPSS 26.0 software, and the test level was set to α = 0.05 (2-tailed test), with *P* < .05 indicating a statistically significant difference.

Descriptive statistics: count data (e.g., gender and marital status) were described by frequency and composition ratio (n, %); measurement data (e.g., age, Readiness for Hospital Discharge Scale and the Discharge Guidance Quality Scale score) were 1st tested for normality using the Shapiro–Wilk test. For data that conformed to a normal distribution, they were described as mean ± standard deviation.

Inferential statistics:

1. For the comparison of the outcome variable (Readiness for Hospital Discharge Scale score) among 2 groups of independent samples, the independent samples *t*-test was used.
2. For the comparison among multiple groups, 1-way analysis of variance was used.

Multiple stepwise regression analysis: to explore the influencing factors of the outcome variable (Readiness for Hospital Discharge Scale score), multiple stepwise regression analysis was conducted with the Readiness for Hospital Discharge Scale total score as the dependent variable and the independent variables (general information items, the Quality of Discharge Teaching Scale total score) with *P* < .05 in the univariate analysis as candidate variables. The entry criterion of variables was α = 0.05, and the elimination criterion was α = 0.10. The collinearity of candidate variables was tested before regression (variance inflation factor < 10 was considered no significant collinearity), and the regression model was evaluated using the adjusted *R*².

## 3. Results

### 3.1. Common method bias test

The method of data collection in this study was a questionnaire survey; therefore, there is a possibility of common method bias. Harman’s 1-way test was used to test the collected data, and the results showed that 9 factors had an eigenroot > 1, and the 1st factor had an explanatory rate of 27.00%, which is lower than the critical standard value of 40%.^[11]^ The results indicated that there was no significant common method bias in the data of this study.

### 3.2. Basic information on patients with colorectal cancer stoma

Among the 231 study subjects included, men accounted for a larger proportion (66.23%); the average age of the study subjects was 65.84 ± 10.39 years old; Han ethnicity accounted for most of the study subjects (91.77%); the majority of the subjects were married (99.14%); the overall level of patients’ literacy was low, with the education level of “primary school and below” accounted for 40.26%; in terms of the length of hospitalization days, 15 to 21 days were the most (40.26%); and the incidence of postoperative complications was 21.21%. Further details are provided in Table 1.

**Table 1:** Differences in readiness for hospital discharge scores among colorectal cancer stoma patients with different general information.

Analysis of factors	Form	Number of cases/composition ratio (%)	Score	*t*/*F*	*P* value	Verify by comparing 2 cases
Gender						
Male		153/66.23	120.36 ± 28.51	0.545	.607	
Female		78/33.77	117.90 ± 30.44	0.545	.607	
Age						
18~50 yr old		17/7.36	138.24 ± 16.18	1.935	.106	
51~60 yr old		47/20.35	117.28 ± 30.22			
61~70 yr old		88/38.09	118.30 ± 31.43			
71~80 yr old		66/28.57	118.08 ± 26.90			
>80 yr old		13/5.63	118.92 ± 28.31			
Nation						
Han		212/91.77	120.68 ± 28.76	1.539	.205	
Mongolian		12/5.19	104.92 ± 34.16			
Hui Islamic ethnic group living across China		6/2.60	106.67 ± 28.13			
The rest		1/0.44	127			
Marital status						
Married		229/99.14	119.33 ± 29.18	0.627	.535	
Unmarried		1/0.43	147.00			
Divorced or widowed		1/0.43	137.00			
Operating state						
In-service		21/9.09	121.76 ± 27.87	0.175	.913	
Retirement		114/49.35	120.31 ± 29.21			
Farmers and pastoralists		77/33.33	117.65 ± 29.33			
Jobless		19/8.23	120.00 ± 31.18			
Residence						
Cities and towns		134/58.01	123.61 ± 26.95	2.475	.014	
Countryside		97/41.99	113.89 ± 31.18			
Living arrangement						
Live alone		1/0.43	147.00	0.944	.440	
Residence with spouse		98/38.53	122.17 ± 29.17			
Residence with children and/or parents		1/0.43	137.00			
Residence with spouse, children, and/or parents		131/56.71	116.84 ± 29.29			
The rest		9/3.90	127.56 ± 26.51			
Educational attainment						
Primary and below		93/40.26	117.80 ± 28.82	1.083	.377	
Junior high school		83/35.93	117.96 ± 30.30			
Senior high school		40/17.32	126.88 ± 26.84			
University and above		15/6.49	119.33 ± 30.30			
Monthly per capita household income						
<1000①		39/16.88	118.92 ± 29.76	2.805	.041	③>②*
1001–3000②		140/60.61	115.99 ± 30.27			
3001~5000③		42/18.18	129.81 ± 23.21			
>5000④		10/4.33	128.30 ± 23.91			
Medical payment methods						
(Be) at one’s own expense		2/0.87	142.50 ± 4.95	0.632	.533	
Residents’ medical insurance		179/76.19	119.20 ± 29.68			
Health insurance at this level		53/22.94	119.74 ± 27.71			
Number of days in hospital						
≤7 d		18/7.79	122.50 ± 27.80	0.601	.615	
8~14 d		61/26.41	121.56 ± 29.74			
15~21 d		93/40.26	120.32 ± 28.68			
≥22 d		59/25.54	115.27 ± 29.90			
Tumor site						
Colon (large intestine)		30/12.99	124.13 ± 27.93	0.928	.355	
Rectum (anatomy)		201/87.01	118.84 ± 29.32			
Nature of the stoma						
At the instant sth happens		148/64.07	120.68 ± 28.79	0.799	.425	
Permanently		83/35.93	117.48 ± 29.81			
Postoperative complication						
Clogged		182/78.79	122.71 ± 26.93	2.865	.006	
Be		49/21.21	107.69 ± 33.93			

*F* = *F*-value for multicategory variables, *t* = *t* value for 2-category variables.

* *P* < .05.

### 3.3. Current status of discharge readiness of colorectal cancer stoma patients

The normality test was performed on the data of Readiness for Hospital Discharge Scale scores of cancer stoma patients. The absolute value of the coefficient of kurtosis of each variable was <10, and the absolute value of the coefficient of skewness was <3, which satisfied normality.^[12]^

The Readiness for Hospital Discharge Scale scores of CRC stoma patients with CRC conformed to a normal distribution and were described using mean ± standard deviation. The total Readiness for Hospital Discharge Scale score of CRC stoma patients was 119.53 ± 29.14, which was moderate. The size of the scores of the 4 dimensions of the Readiness for Hospital Discharge Scale was in the following order: anticipatory support > knowledge of the disease > personal status > coping ability. Further details are provided in Table 2.

**Table 2:** Readiness for Hospital Discharge Scale scores of patients with colorectal cancer stoma.

Sports event	Score	Entry parity (accountancy)	Arrange in order
Readiness for hospital discharge	119.53 ± 29.14	6.64 ± 1.62	–
Personal status dimension	19.97 ± 6.00	6.658 ± 2.00	3
Disease knowledge dimension	53.27 ± 15.10	6.659 ± 1.89	2
Response capacity dimension	19.62 ± 6.07	6.54 ± 2.02	4
Expected support dimensions	26.66 ± 7.90	6.664 ± 1.98	1

– = no data.

### 3.4. Analysis of factors influencing discharge readiness in colorectal cancer stoma patients

#### 3.4.1. Differences in discharge readiness scores of colorectal cancer stoma patients on general information

The results showed that the differences in readiness to discharge scores of cancer stoma patients according to the place of residence, per capita monthly family income, and postoperative complications were statistically significant (*P* < .05), as presented in Table 1.

#### 3.4.2. Multiple stepwise regression analysis of discharge readiness in colorectal cancer stoma patients

To further understand the factors influencing discharge readiness in CRC stoma patients, multiple stepwise regression analyses were performed using the *t*-test and 1-way analysis of variance statistically significant variables (current address, monthly per capita household income, and postoperative complication), the quality of discharge guidance score as the independent variable, and the readiness to discharge score of CRC stoma patients as the dependent variable. The results showed that the influences that eventually entered the regression model were the quality of discharge instructions, postoperative complications, and place of residence, as detailed in Table 3.

**Table 3:** Multiple stepwise regression analysis of discharge readiness scores of 231 colorectal cancer stoma patients.

Independent variable	Unstandardized coefficient		Standardised coefficient	*t* value	*P* value	Covariance statistics	
	*B*	Standard error	*β*			Tolerances	VIF
Constant	120.757	11.127	–	10.852	<.001	–	–
Quality of discharge teaching	0.208	0.060	0.218	3.476	<.001	0.989	1.011
Postoperative complication	−14.042	4.455	−0.197	−3.152	.002	0.994	1.006
Current address	−7.553	3.709	−0.128	−2.036	.043	0.984	1.016

*F* = 39.738, *P* < .001, *R*^2^ = 0.114, adjusted *R*^2^ = 0.102, – = blank term.

VIF = variance inflation factor.

## 4. Discussion

This study showed that the total Readiness for Hospital Discharge Scale score of CRC stoma patients was 119.53 ± 29.14, which was moderate. It failed to reach the mean postoperative discharge readiness score for adults measured by Weiss et al^[13]^: slightly higher than the results of a cross-sectional study by Qu et al^[14]^ on 127 elderly CRC patients with colostomy (108.02 ± 16.89), which may be related to the overall older age of the study population by Qu et al, and slightly lower than the results of a cross-sectional study (131.39 ± 29.41) by Fan et al.^[15]^ for 119 CRC colostomy patients, which may be related to the regional economy and medical technology level.

Among the 4 dimensions measured by the Readiness for Hospital Discharge Scale, the highest score is the expected support dimension, which is consistent with the results of the study by Chen et al,^[16]^ which may be related to the fact that in our cultural context, the main companions during hospitalization are family members, and patients are able to obtain sufficient support from their families. The lowest score is the coping ability dimension, which is consistent with the results of the study by Zhao et al,^[17]^ which is not difficult to explain, with increases in age, cognitive decline, and risk of incapacitation increase in the elderly, which directly leads to a decline in coping ability,^[18]^ and the care of the colostomy is a series of difficult professional operations, the patient has difficulties in the adaptation of the stoma and coping with the stoma care operations, which leads to poor scores in the coping ability dimension.

The survey suggested that the quality of discharge instructions might be an influential factor in the discharge readiness of cancer stoma patients (*P* < .001), which was in line with the results of the Nurhayati et al’s^[19]^ study. It could be that high-quality discharge instructions not only help patients acquire correct nursing skills and knowledge but also effectively reduce their psychological burden, enhance their self-efficacy, and promote their positive adaptation to postoperative life. Meanwhile, discharge instructions might also help patients transition more smoothly to an independent life after discharge by enhancing their ability to utilize social support, improving their ability to care independently, and reducing the risk of complications. Therefore, the impact of the quality of discharge instructions on patients’ readiness to be discharged should not be ignored, and detailed discharge instructions, especially for knowledge of enterostomies and management of complications, may help patients implement discharge instructions.

The findings indicated that postoperative complications might have an impact on the discharge readiness of cancer stoma patients (*P* < .05), which was consistent with the results of studies by Wei et al^[20]^ and Scheffel et al.^[21]^ Common colostomy complications include peristoma dermatitis, stoma stenosis, stoma prolapse, parastomal hernia, and stoma leakage, which could result in unpleasant experiences such as pain and itching and even frustrate the confidence of patients and their primary caregivers in stoma care, leading to a lower readiness for discharge.

The results of the study implied that the place of residence might be an influencing factor on the discharge readiness of CRC stoma patients (*P* < .05), which was consistent with the results of the study conducted by Yu^[10]^ and might be related to the convenience of access to healthcare resources. It was possible that convenient access to healthcare for patients living in towns and cities and the popularity of community hospitals had also led to a reduction in the cost of access to healthcare and the convenience of purchasing stoma care tools, which might have a positive effect on readiness for discharge.

### 4.1. Shortcomings of this study

A limitation of this study is that data were collected by the researchers themselves, which may lead to data collection bias. For subsequent studies, data collection could be entrusted to 3rd-party institutions or independent researchers who were not involved in the study design and hypothesis formulation. In this way, data collectors would be unaware of the research purposes and core hypotheses, thereby avoiding the influence of their subjective tendencies on data collection.

## 5. Conclusion

The discharge readiness of CRC stoma patients is moderate and requires further improvement. Among them, the quality of discharge instructions, postoperative complications, and place of residence were the key factors influencing the discharge readiness of CRC stoma patients with CRC. It is suggested that healthcare professionals should pay attention to special patients and develop interventions according to the relevant influencing factors to improve the readiness of CRC stoma patients to be discharged from the hospital and successfully complete the transition from hospital to home.

## Author contributions

**Conceptualization:** Shuhan Li, Shu-Han Li.

**Data curation:** Shuhan Li, Shu-Han Li.

**Formal analysis:** Shuhan Li, Shu-Han Li.

**Project administration:** Wen-Nv Hao.

**Resources:** Kai-Xin Liu.

**Software:** Kai-Xin Liu.

**Writing – original draft:** Shuhan Li, Shu-Han Li.

**Writing – review & editing:** Wen-Nv Hao.
